# Impact of smoking exposure on human papillomavirus clearance among Chinese women: A follow-up propensity score matching study

**DOI:** 10.18332/tid/161026

**Published:** 2023-03-20

**Authors:** Kangli Ma, Shu Li, Sufang Wu, Jingfen Zhu, Yongbin Yang

**Affiliations:** 1 Department of Obstetrics and Gynecology, Shanghai General Hospital, Shanghai Jiao Tong University School of Medicine, Shanghai, People’s Republic of China; 2 School of Public Health, Shanghai Jiao Tong University, Shanghai, People’s Republic of China

**Keywords:** human papillomavirus, smoking, persistent infection, normal uterine cervix

## Abstract

**INTRODUCTION:**

Smoking has been proven to increase the risk of cervical cancer, but it is still controversial whether smoking reduces women’s ability to clear human papillomavirus (HPV) infection. This study investigated the association between smoking behaviors during follow-up and clearance of HPV infection in women with HPV-positive and pathologically normal uterine cervix in China, using a propensity score matching (PSM) analysis.

**METHODS:**

The present prospective study included data from women examined in the Gynecology Department of Shanghai General Hospital from January 2018 to June 2020. Twenty patients who smoked throughout follow-up were selected and matched with 60 patients using the 1:3 PSM method on age, marital status, and whether infected with high-risk HPV (HR-HPV). At each visit, smoking and sexual behaviors were collected. The Kaplan–Meier method and a Cox proportional hazard regression model were used to evaluate the probability of clearing HPV infection within a 2-year follow-up.

**RESULTS:**

A total of 80 patients were included in the study, all of whom were infected with at least one HR-HPV type at baseline. Current smokers had a lower likelihood of clearing the HPV infection than current non-smokers, after adjusting for a history of sexually transmitted diseases (STD), HPV infection status, and sexual behaviors during follow-up (AHR=0.478; 95% CI: 0.239–0.958, p=0.037). Additionally, longer duration, higher frequency and larger doses of smoking correlated with the lower clearance possibility of HPV infection (p for trend=0.029, 0.022 and 0.026, respectively).

**CONCLUSIONS:**

This study showed that the use of tobacco throughout follow-up could increase the risk of a persistent HPV infection, this risk being higher for smokers with heavier tobacco consumption. Our results should alert HPV-positive women to reiterate the advice to cut-back on or stop smoking.

## INTRODUCTION

Cervical cancer remains the fourth most common cancer among women worldwide, with approximately 604000 new cases and 341000 deaths in 2020^1^. Unfortunately, over 85% of the global cervical cancer burden occurs in low-income and middle-income countries^2^, with China and India having the heaviest share^3^. Even though cervical cancer screening programs and HPV vaccination have reduced the incidence and mortality globally, China’s situation is fraught with difficulties, and mortality has even increased in recent years^4^.

Human papillomavirus (HPV) infection is a common sexually transmitted disease. Most HPV infections are transient and disappear within 12–24 months after infection^5^. Persistent HPV infection, especially of high-risk types (including types 16, 18, 31, 33, 34, 35, 39, 45, 51, 52, 56, 58, 59, 66, 68, and 70), is considered the necessary cause of almost all cervical lesions and cancer progressions, but this is not the sufficient condition of cervical cancer^6^. Besides, studies have linked cigarette smoking, even passive smoking, to an increased risk of cervical cancer or its precursor lesions^7^. Smoking seems to be the most important risk factor, except for HPV infection, for cervical disease.

The exact results demonstrating the precise impact of smoking on HPV persistence and the corresponding mechanism, however, have not been fully established. Smoking was linked to persistent HPV infection in a study of Romanian women (OR=2.320, p=0.033)^8^. Another study in Britain found no significant connection between smoking and the probability of HR-HPV persistence after six months (risk ratio, RR=1.12; p=0.516)^9^. Nevertheless, similar research was rarely conducted in women with a normal pathological cervical biopsy.

It is reported that 341 million (30%) tobacco smokers lived in China in 2019^10^. Although the most active smokers are men, women also contributed. Smoking prevalence has reached 3.54% (2.91%–4.18%) among Chinese women^10^. And in China, the smoking rate among young women (aged 15–24 years) increased by 74.0% from 1990 to 2019^11^.

Women with HPV-positive and biopsy-negative results are recommended to have at least a one-year follow-up without the requirement for treatment, according to the American Society for Colposcopy and Cervical Pathology (ASCCP)^12^. Focusing on the natural history of HPV infection in these individuals can allow us to eliminate the distraction of treatments. Our purpose in this analysis was to determine whether there was a correlation between smoking behaviors during follow-up and clearance of HPV infection in women with pathologically normal uterine cervix.

## Methods

### Study design and population

**Figure 1:**
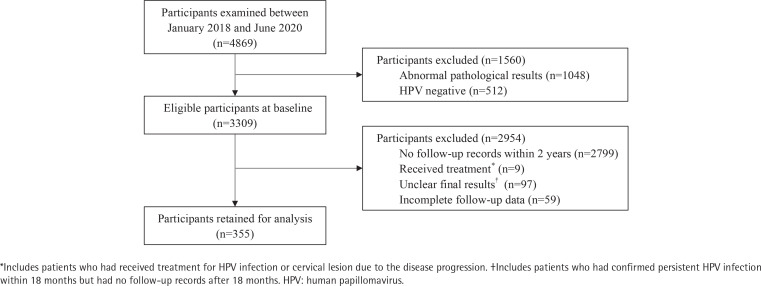
Participants selection process

This study was a prospective non-intervention study designed to investigate the natural history of HPV infections in women and its relationship with smoking status and behaviors during follow-up. The clinical data of 4869 patients examined for cervical pathological biopsy in the Gynecology Department of Shanghai General Hospital were assessed between January 2018 and June 2020. All cases provided results of HPV genotyping, cytological examination and cervical biopsy at baseline, among which 3309 cases were HPV-positive and pathologically normal. Figure 1 demonstrates details on participant selection process. After the signature of an informed consent form at the baseline visit, every participant completed a risk factor questionnaire consisting of age, marital status, residency, education level, history of sexually transmitted diseases (STD), age at first intercourse, number of lifetime sexual partners, and HPV vaccination.

Follow-up visits were scheduled at intervals of six to twelve months after the cervical biopsy. Infections were followed until the clearance, or until the last visit if the participant remained positive throughout the whole period, or until a diagnosis of higher lesions, which means the follow-up would be interrupted by treatment. At each follow-up visit, patients not only were retested for HPV DNA and genotype, but also completed a brief questionnaire on relevant gynecological events and epidemiological features since the last visit, mainly about smoking and sexual behaviors. According to the inclusion or exclusion criteria, 355 patients were chosen for analysis, among which 20 patients smoked during follow-up.

In our study, clearance of HPV infection was defined as when none of the types from the first HPV detection was detected at the subsequent visit. The estimated time to clearance was based on the date of the first negative visit. Persistent HPV infection meant the presence of the same HPV genotype detected at enrollment on one or more consecutive examinations at subsequent visits.

Inclusion criteria were: 1) positive for one or more HPV types diagnosed in Shanghai General Hospital at baseline; and 2) colposcopy and cervical biopsy were performed in the same hospital and confirmed normal at baseline. Exclusion criteria were: 1) no follow-up records within two years; 2) underwent any treatment to clear HPV infection during follow-up; 3) unclear final results owing to lack of follow-up; 4) previous hysterectomy or conization; and 5) with other malignant tumors.

### Questionnaires related to smoking behaviors

Information on current smoking behaviors was collected using a standardized questionnaire at each follow-up visit. All patients were classified into current smokers and current non-smokers based on these questionnaires. Current smokers were defined as those women who smoked during follow-up, including regular smoking and occasional tobacco use, while the others were considered current non-smokers. To further analyze the relationship between smoking behaviors and the capacity to clear HPV infection, the duration (years of smoking), frequency (days of smoking per month), and dose (numbers of cigarettes smoked per day) were also collected.

To facilitate analysis and discussion, patients were grouped based on the duration, frequency and dose of tobacco use. Participants were divided into three groups based on their smoking duration: current non-smokers, those who had smoked for 1–10 years, and those for >10 years. Similar limits were used for grouping based on frequency and dose, such as 15 days per month and 10 cigarettes per day.

### HPV detection and typing methods

Testing for HPV was conducted by using a PCR-reverse dothybridization technology, with the help of a PCRRDB HPV genotyping assay (Yaneng Bioscience Co., Ltd., China)^13^; 23 HPV types can be detected in total, covering 17 HR-HPV types (16, 18, 31, 33, 35, 39, 45, 51, 52, 53, 56, 58, 59, 66, 68, 73, 82) and 6 low-risk HPV types (6, 11, 42, 43, 81, 83). DNA extraction, PCR amplification and result analysis were all conducted according to the manufacturer’s instructions.

### Colposcopy and pathological examination

Colposcopies were performed when HPV detection or cytological results were abnormal. The cervical tissue was obtained from a colposcopy biopsy. Suspicious lesions were stained with acetic acid and iodine reagent, and then biopsied on the basis of colposcopy images. The samples were processed with standard histopathological methods and evaluated by at least two pathologists. All colposcopy operations were performed by qualified colposcopy specialists in our center. The pathology results were classified as normal, cervical intraepithelial neoplasia CIN1, CIN2, CIN3, or carcinoma *in situ*.

### Statistical analysis

In order to control for the differences in baseline characteristics between smoking and non-smoking groups, we performed propensity score matching (PSM) using the nearest neighbor matching algorithm. Propensity scores of individuals were calculated based on logistic regression analysis with a caliper width of 0.1. Variables used for matching were age, marital status, and whether infected with HR-HPV. Ratio was paired at 1:3 for patients who smoked versus those who never smoked during follow-up, and another 60 eligible participants were identified after matching. Standardized mean difference (SMD) was used to evaluate the balance of the observed covariates across groups before and after matching. Optimal balance on a parameter is generally achieved when the SMD is ≤0.1.

The chi-squared test and t-test were used to assess the differences in the distributions of categorical and continuous variables, respectively, between current smokers and current non-smokers. The survival curves of clearing during the follow-up were calculated using the Kaplan-Meier method and compared with the log-rank test. Univariate analyses were conducted to assess factors associated with the clearance of HPV infection. The final multivariable model, a Cox proportional-hazards model, was fitted with a subset of risk variables to control for extra potential confounding effects caused by the women’s characteristics. We estimated the hazard ratios (HR) and corresponding 95% confidence intervals (CIs). The value for p-trend was used to further assess whether heavier smoking was linked to more persistent HPV infection, based on smoking data of duration, frequency and dose. Two-sided p<0.05 values were considered to be statistically significant. All statistical analyses were performed using software package of social science statistical software version 26 (SPSS, IBM Co., Armonk, NY, USA).

## Results

### Sociodemographic characteristics between smokers and non-smokers

We analyzed the basic demographic information from enrollment and follow-up data before and after matching (Table 1). Most variables were more balanced after PSM, though some differences remained. After matching, all women were infected by HR-HPV at baseline, and a total of 154 type-specific infections were detected in these 80 participants. The mean age of current smokers and current non-smokers was 34.40 years (median=33; range: 22–63) and 34.40 years (median=34; range: 21–63), respectively. Among HPV-positive women, current smokers were significantly more likely to be infected with more than one HPV type (p=0.038). Apart from this, there was no statistically significant difference in terms of age, marital status, residency, education level, HPV infection status, history of STD, age at first intercourse, number of lifetime sexual partners, HPV vaccination or sexual behaviors during follow-up, between current smokers and current non-smokers (p>0.05).

**Table 1:** Distributions of epidemiological and follow-up characteristics between current smokers and current non-smokers before and after matching

	*Unmatched participants*				*Matched participants*			
*Characteristics*	*Current smokers (N=20) n (%)*	*Current non-smokers (N=335) n (%)*	*SMD*	p*	*Current smokers (N=20) n (%)*	*Current non-smokers (N=60) n (%)*	*SMD*	p*
**Age** (years)								
Mean ± SD	34.40 ± 10.35	44.7 ± 13.28	-0.87	0.016	34.40 ± 10.35	34.40 ± 9.62	0.00	0.719
≤35	13 (65.0)	103 (30.7)	0.73	0.002	13 (65.0)	41 (68.3)	-0.07	0.783
>35	7 (35.0)	232 (69.3)			7 (35.0)	19 (31.7)		
**Marital status**								
Single, never married	8 (40.0)	46 (13.7)	0.62	0.020	8 (40.0)	24 (40.0)	0.00	1.000
Married without child	0	21 (6.3)	-0.37		0	0		
Married with children	11 (55.0)	256 (76.4)	-0.46		11 (55.0)	33 (55.0)	0.00	
Divorced	1 (5.0)	12 (3.6)	0.07		1 (5.0)	3 (5.0)	0.00	
**Residence**								
Rural	8 (40.0)	85 (25.4)	0.32	0.148	8 (40.0)	20 (33.3)	0.14	0.588
Urban	12 (60.0)	250 (74.6)			12 (60.0)	40 (66.7)		
**Education level**								
≤High school	9 (45.0)	24 (7.2)	0.95	0.000	9 (45.0)	18 (30.0)	0.31	0.431
Some college/ vocational	5 (25.0)	217 (64.8)	-0.87		5 (25.0)	16 (26.7)	-0.04	
≥College	6 (30.0)	94 (28.0)	0.04		6 (30.0)	26 (43.3)	-0.28	
**Sexual life during follow-up**								
Active	16 (80.0)	213 (63.6)	0.37	0.136	16 (80.0)	48 (80.0)	0.00	1.000
Never	4 (20.0)	122 (36.4)			4 (20.0)	12 (20.0)		
**HPV infection status**								
Multiple	13 (65.0)	148 (44.2)	0.43	0.069	13 (65.0)	23 (38.3)	0.55	0.038
Single	7 (35.0)	187 (55.8)			7 (35.0)	37 (61.7)		
**History of STD**								
Yes	4 (20.0)	14 (4.2)	0.50	0.009	4 (20.0)	9 (15.0)	0.13	0.861
Never	16 (80.0)	321 (95.8)			16 (80.0)	51 (85.0)		
**Age at first intercourse** (years)								
≤20	11 (55.0)	96 (28.7)	0.55	0.013	11 (55.0)	26 (43.3)	0.24	0.365
>20	9 (45.0)	239 (71.3)			9 (45.0)	34 (56.7)		
**Number of lifetime sexual partners**								
≤3	13 (65.0)	302 (90.1)	-0.63	0.002	13 (65.0)	48 (80.0)	-0.34	0.288
>3	7 (35.0)	33 (9.9)			7 (35.0)	12 (20.0)		
**HPV vaccination**								
Has been vaccinated	1 (5.0)	34 (10.1)	-0.19	0.716	1 (5.0)	10 (16.7)	-0.38	0.349
Never	19 (95.0)	301 (89.9)			19 (95.0)	50 (83.3)		

* Categorical and continuous variables were analyzed by chi-squared test and t-test, respectively. Statistical significance at p<0.05.

HPV: human papillomavirus. STD: sexually transmitted diseases. SMD: standardized mean difference.

For all the participants, the mean duration of follow-up was 19.2 months (median=18.6; range: 6–39), with an average of 2.5 visits (median=2.0). None of the participants received treatment associated with HPV clearance throughout follow-up. Within two years of follow-up, 56 patients cleared their HPV infection spontaneously, including 10 of 20 current smokers and 46 of 60 current non-smokers. In contrast, 24 individuals were found to have persistent HPV infection by the end of 2 years.

### Associations between smoking behaviors and HPV clearance

**Figure 2:**
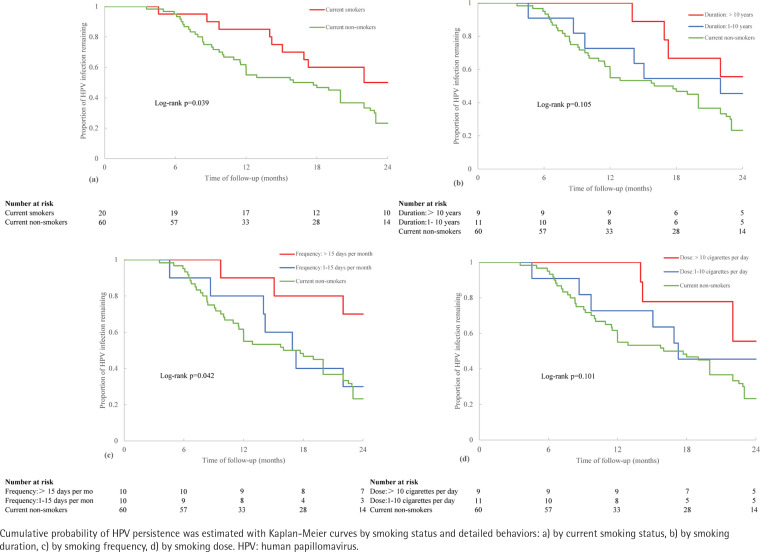
Risk of HPV infection persistence in relation to smoking behaviors within a 2-year follow-up

Figure 2 presents the univariate clearance (Kaplan-Meier) curves for the cumulative probability of clearing HPV infection within 2 years of follow-up among groups classified by current smoking behaviors. Compared to current non-smokers, HPV infection was significantly less likely to be cleared among current smokers (log-rank test: p=0.039, Figure 2a). In addition, the frequency of tobacco intake monthly was also positively connected with a poorer HPV infection clearance (log-rank test: p=0.042, Figure 2c). However, the duration of smoking (log-rank test: p=0.105, Figure 2b) and dose of tobacco use every day (log-rank test: p=0.101, Figure 2d) were shown to have no significant influence on the clearance in the patients.

To select covariates statistically associated with the risk of persistent HPV infection, we used univariate Cox regression method to analyze 10 factors (Table 1). None was found to have influence on persistent HPV infection in our cases. To better evaluate the association between smoking behaviors and HPV infection clearance in those patients, a multivariable Cox proportional hazard regression model was performed, with adjustments for HPV infection status, history of STD and sexual behaviors during follow-up (Table 2). We also estimated the unadjusted hazard ratio (HR) and adjusted hazard ratio (AHR).

**Table 2:** Association between current smoking behaviors and clearance of HPV infection within a 2-year follow-up after matching

*Current smoking behaviors*	*n*	*HP infection clearance*	Hazard ratio (HR) and adjusted hazard ratio (AHR)* for HPV infection clearance			
			*HR (95% CI)*	*p*	*AHR (95% CI)*	*p*
**Status**						
Current non-smokers (Ref.)	60	48	1		1	
Current smokers	20	10	0.499 (0.251–0.989)	0.046	0.478 (0.239–0.958)	0.037
**Duration** (years of smoking)						
0 (Ref.)	60	48	1		1	
1–10	11	6	0.603 (0.258–1.414)	0.245	0.589 (0.248–1.395)	0.229
>10	9	4	0.395 (0.142–1.100)	0.075	0.360 (0.128–1.013)	0.053
				p for trend=0.043		p for trend=0.029
**Frequency** (days of smoking per month)						
0 (Ref.)	60	48	1		1	
1–15	10	7	0.848 (0.383–1.880)	0.685	0.676 (0.294–1.555)	0.356
>15	10	3	0.254 (0.079–0.817)	0.021	0.254 (0.087–0.910)	0.034
				p for trend=0.020		p for trend=0.021
**Dose** (numbers of cigarettes smoked per day)						
0 (Ref.)	60	48	1		1	
1–10	11	6	0.621 (0.265–1.454)	0.272	0.622 (0.263–1.473)	0.281
>10	9	4	0.385 (0.138–1.071)	0.067	0.343 (0.122–0.964)	0.042
				p for trend=0.040		p for trend=0.026

Univariable and multivariable Cox regression models were used to evaluate the correlations.

* Adjusted for history of sexually transmitted diseases (STD), HPV infection status, and sexual behaviors during follow-up.

HPV: human papillomavirus.

Based on the HR and corresponding p-value, the duration, frequency, and dose (p for trend=0.043, 0.020, and 0.040, respectively), as well as the current status of smoking (HR=0.499; 95% CI: 0.251–0.989, p=0.046), were all significantly linked with HPV persistence. The probability of HPV clearance was lower among current smokers than among current non-smokers, even after adjusting for those aforementioned covariates (AHR=0.478; 95% CI: 0.239–0.958, p=0.037). Furthermore, longer smoking duration, more frequent smoking, and larger doses of tobacco all had a negative correlation with the likelihood of clearing an HPV infection (p for trend=0.029, 0.022 and 0.026, respectively). It was convincingly demonstrated that the risk of a persistent HPV infection was higher for smokers with heavier tobacco consumption.

## DISCUSSION

To our knowledge, the present study is the first to examine the association between status and specific behaviors of smoking during follow-up and clearance of HPV infection among women with pathologically normal uterine cervix. Previous studies mainly included HPV-positive and cytologically normal participants at recruitment^14-17^, while some paid attention to those with low-grade or equivocal cytological abnormalities^18,19^. In terms of this association, until now, there has not been any published research focusing on the pathological findings of cervical biopsies. A study carried out in women with normal cytology in Daqing, a city in China, has found that cigarette smoking was not correlated with type-specific HPV persistence^17^. However, cytology is known to have a poor sensitivity of approximately 53.0%^20^. Part of the reasons are that cytological results could be affected by the quality of the sample, the professional ability and the mental state of doctors. And the diagnosis of cervical diseases in all patients depended on cervical histopathology. Compared to the cytology examination, we could demonstrate more accurately that our participants were free of cervical lesions. In our cases, we excluded women with biopsy-positive lesions at baseline from the analysis, aiming to eliminate the interference of any related treatment.

In the present study, the 1:3 PSM method was used to match current non-smokers with current smokers to control for possible confounding effects at baseline. After matching, our univariate analysis demonstrated an increased risk for HPV infection persistence in women who smoked compared with those who never smoked during follow-up. Multivariable analysis showed the same results after adjusting for HPV infection status, history of STD and sexual behaviors during follow-up. In addition, we detected a more complex relationship between smoking for a longer time, more often, and at larger doses with a lower likelihood of clearing HPV infection within a 2-year follow-up. These findings indicate that smoking influenced the natural history of HPV infections in women, implying the beneficial effects of smoking reduction and cessation.

As a well-known risk factor for the progression of cervical lesions with HPV infection, smoking was supposed to promote a persistent infection. However, the association has never been clearly evaluated, and some previous studies have found negative^21^ or null^9,15-18,22^ associations between current smoking and HPV persistent infection. The inconsistency of these studies may be due to different definitions of HPV persistence or clearance, sample sizes, follow-up intervals and durations and so on. A US-based study among young women reported that smoking was protective against persistent HPV infection, but researchers were unsure whether it was because of a biological or a confounding effect^21^. In another UK-based study, no association between smoking and persistent HR-HPV infection was found, partly on account of the short 6-month follow-up^9^.

Our results are in accordance with the majority of published studies in this field^8,14,23-25^ and the well-accepted advice to quit smoking during follow-up^26^, indicating that smoking plays an important role in the process of persistent HPV infection in women. Among these studies, only a study based on women aged 18–35 years detected a significant relationship with increasing duration of years of smoking, but not with smoking intensity^23^.

It is worth noting that few studies further explored the correlation between smoking behaviors and HPV infection persistence in the different subgroups. A study based on Korean women found that among alcohol drinkers, women exposed to secondhand smoking at home or in the workplace were at high risk of both 1-year HR-HPV persistence and 2-year HR-HPV persistence. However, among non-alcohol drinkers, there was no association between smoking or secondhand smoking status and these risks^19^. Another study, based on women in the US, indicated that only among HIV-seronegative women the clearance rate for HR-HPV infections was notably lower in ever smokers than in never smokers^27^.

Although the precise mechanism of how smoking influences HPV infection persistence is not clearly understood, there are some speculations. This correlation is more likely to be explained by the effect of smoking behaviors on the immune system rather than its DNA damage, partially because persistence is greatly influenced by host immunity. This hypothesis is also supported by the fact that in about 80% of HPV-infected women, HPV clearance is a rapidly evolving event and occurs before viral integration into the host genome^28^. The non-significant effect of smoking on the detection of E7 protein, an indicator of later stages in the natural history of the infection, also implied that smoking has a more prominent role in earlier stages^29^. Therefore, the ability of women to clear HPV infections seems to be related to the host immune response.

Smoking has been proven to cause a wide range of suppression of immunological functions^23^. In smokers compared with non-smokers, a significant reduction of Langerhans cells in the normal cervical epithelium was observed^30^. This change could lead to local immunosuppression and facilitate the establishment and persistence of local HPV infection because Langerhans cells are essential in detecting and presenting viral antigens to T cells. It was further found that smoking showed direct inhibiting effects on T cells, increasing the percentage of CD8^+^ T cells while lowering CD4^+^ T cells^31^. As for humoral immune responses, smokers are deemed to have an increased risk of persistent HPV infection by reducing antibodies or preventing the development of antibodies^32^. In addition, smoking can decrease the activity of the natural killer cells, affecting the innate immune system^33^. Combined, it has been generally established that current smoking is associated with a decreased capability of the immune system, thus hindering HPV clearance in the cervix.

Besides suppressing the immune system, smoking is likely to contribute to persistent HPV infection in other ways. Some confounding effects caused by different lifestyles or characteristics that are different in smokers compared with non-smokers may contribute to additional risk to the persistent infection of HPV. For example, more liberal sexual habits and risky sexual behaviors were more familiar among smokers^34^, though this phenomenon was not seen in our study. Higher HPV 16 and HPV 18 DNA load was also associated with current smokers^35^, while the viral load was inversely associated with clearance^36^. Unfortunately, we did not have information on viral load.

Furthermore, considering persistent HR-HPV infection was the most critical factor of cervical lesions and related cancer^6^, the present study might offer a new thought about how smoking contributes to the progression of cervical cancer. As reported previously, tobacco smoke contains carcinogens that could have a direct transformation effect on the cervix, allowing progress to cancer^7^. Data from the present study indicate that part of the increased risk of progression to disease carried by smoking might be due to its impact on HPV infection persistence and the natural history of HPV infection.

### Strengths and limitations

This study is the first to examine the correlation between smoking behaviors and HPV infection clearance in women with pathologically normal uterine cervix. This strategy of selecting the participants based on cervical biopsy results was more precise and sensitive than cytology.

Nevertheless, our study has several limitations that may have affected the conclusion. First, all patients were diagnosed and followed in a single center, and the restricted sample size did not allow us to conduct subgroup analyses further. Owing to the small number of cases, we gave up assessing the correlation between smoking and persistent infection of different HPV genotypes. Second, some factors were not included in our study. We did not take the specific information on secondhand smoke intake, sleep quality or use of condoms into account since more than half of these women had not completed related content in the questionnaire. We also did not measure viral load at baseline or follow-up. Third, data were based on self-reports from participants who may underreport or overreport their behaviors or attitudes. Our study also lacked a validated biomarker for measuring smoking exposure, such as urine cotinine levels. We were considering introducing this examination at subsequent follow-up visits. As a result, further research is still required to improve these weaknesses.

## Conclusions

This is the first study to report the association between smoking behaviors during follow-up and HPV clearance in women with normal pathological results of cervical biopsy. The significant finding of this study was that smoking during follow-up is associated with a decreased capacity to clear HPV. Additionally, a reduced likelihood of HPV infection clearance was linked to smoking for longer, more frequently, and at larger doses. Although physicians tend to provide no treatment for women who are HPV-positive and pathologically normal, tobacco use cessation may lead to a faster rate of HPV clearance. Further large-scale and long-term studies are needed to assess these associations in order to assess the future burden of cervical cancer, notably in developing countries.

## Data Availability

The data supporting this research are available from the authors on reasonable request.
